# SD-YOLOv8: An Accurate *Seriola dumerili* Detection Model Based on Improved YOLOv8

**DOI:** 10.3390/s24113647

**Published:** 2024-06-04

**Authors:** Mingxin Liu, Ruixin Li, Mingxin Hou, Chun Zhang, Jiming Hu, Yujie Wu

**Affiliations:** 1 School of Electronics and Information Engineering, Guangdong Ocean University, Zhanjiang 524088, China; liumx@gdou.edu.cn (M.L.); 2112210031@stu.gdou.edu.cn (C.Z.); 2112210006@stu.gdou.edu.cn (J.H.); 2 Guangdong Provincial Key Laboratory of Intelligent Equipment for South China Sea Marine Ranching, Zhanjiang 524088, China; 3 Naval Architecture and Shipping College, Guangdong Ocean University, Zhanjiang 524088, China; 2112212010@stu.gdou.edu.cn (R.L.); 2112312017@stu.gdou.edu.cn (Y.W.); 4 School of Mechanical Engineering, Guangdong Ocean University, Zhanjiang 524088, China

**Keywords:** *Seriola dumerili*, attention mechanism, YOLOv8, deformable convolution, small object detection

## Abstract

Accurate identification of *Seriola dumerili* (SD) offers crucial technical support for aquaculture practices and behavioral research of this species. However, the task of discerning *S. dumerili* from complex underwater settings, fluctuating light conditions, and schools of fish presents a challenge. This paper proposes an intelligent recognition model based on the YOLOv8 network called SD-YOLOv8. By adding a small object detection layer and head, our model has a positive impact on the recognition capabilities for both close and distant instances of *S. dumerili*, significantly improving them. We construct a convenient *S. dumerili* dataset and introduce the deformable convolution network v2 (DCNv2) to enhance the information extraction process. Additionally, we employ the bottleneck attention module (BAM) and redesign the spatial pyramid pooling fusion (SPPF) for multidimensional feature extraction and fusion. The Inner-MPDIoU bounding box regression function adjusts the scale factor and evaluates geometric ratios to improve box positioning accuracy. The experimental results show that our SD-YOLOv8 model achieves higher accuracy and average precision, increasing from 89.2% to 93.2% and from 92.2% to 95.7%, respectively. Overall, our model enhances detection accuracy, providing a reliable foundation for the accurate detection of fishes.

## 1. Introduction

*Seriola dumerili* (SD), commonly known as “Kanpachi”, is a fish species primarily found in the upper layers of the ocean. It inhabits tropical and temperate waters of major ocean regions [1]. Species of the genus *Seriola* are targeted for aquaculture due to their fast growth and large size. Among them, *S. dumerili* is distributed in tropical seas and is actively farmed in the Mediterranean Sea and Asian seas [2,3,4], holding significant value in both commercial and recreational fisheries. Its firm and flavorful flesh makes it a prized target for various culinary applications, including in baking, in grilling, and as sashimi [5]. In the complex underwater environment, conventional aquaculture practices heavily rely on artificial observation and experimental judgment [6,7]. These methods are particularly challenging when dealing with schools of fish, where misdetection and missed detections are common occurrences. The limitations of human observation extend to the difficulty in accurately identifying both nearby and distant fish as well as small fish, leading to low management effectiveness and the formulation of incorrect strategies in aquaculture [8]. The need for intelligent and accurate detection of fish underwater is paramount for advancing fish behavior research, implementing intelligent feeding strategies, and monitoring fish health and diseases. To address these challenges, this paper introduces an improved detection model designed to be robustly applied to the task of underwater fish recognition.

Fish recognition tasks are particularly challenging compared to the identification of other terrestrial organisms [9,10,11]. First, dataset acquisition is difficult due to the stringent requirements for collection devices in underwater environments. The existing publicly available datasets, such as LifeCLEF15 [12], Fish4Knowledge [13], LSCD1 [14], and FishPak [15], are often plagued by image noise, blurriness, and suboptimal lighting conditions. Second, accurately capturing the texture, color, and shape information of fish within complex aquatic environments is a daunting task. Traditional fish recognition methods depend on feature extraction for target classification. For example, Fouad et al. [16] utilized support vector machines alongside the scale-invariant feature transform (SIFT) and speeded-up robust features (SURF) algorithms for feature extraction to classify Nile tilapia based on local features. Ravanbakhsh et al. [17] employed Haar features in conjunction with principal component analysis (PCA) for the classification of southern bluefin tuna in aquaculture settings. Bilal et al. [18] adopted the centroid–contour distance method for classifying fish species with dual dorsal fins. Cutter et al. [19] implemented a cascade of Haar features for the detection and recognition of benthic fish during unconstrained underwater surveys. Dhawal and Chen [20] generated representative feature vectors through the use of a histogram of oriented gradients (HOG) and color histograms for the identification of 10 similar fish species. While these appearance-based techniques have shown commendable detection performance in static images, they require considerable human effort in feature design and are less robust in adapting to dynamic and complex underwater environments, exacerbated by limited data availability.

The advent of deep learning has ushered in new avenues for fish object detection. Deep learning techniques can autonomously extract both low- and high-level features from extensive datasets. The inherent complexity of deep learning, with its layered architectures and self-learning algorithms, allows them to capture intricate details that may elude traditional detection methods, thus enhancing the overall accuracy and adaptability of fish recognition systems in diverse aquatic environments. For instance, Li et al. [21] designed an automated fish recognition system using the Fast R-CNN framework, achieving an average recognition accuracy of 81.2% across 24,277 images of 18 fish species. Notably, the system required only 4 h to train and processed individual images in 0.311 s, indicating a substantial enhancement in the efficiency of fish object detection. In another study, Salman et al. [22] leveraged a mixture of Gaussian mixture models (GMMs) and optical flow to isolate initial motion features from video footage of fish. These motion-centric features, combined with texture and shape data from the original frames, informed the training of the R-CNN network. This approach achieved detection accuracies of 87.44% and 80.02% on the Fish4Knowledge and LifeCLEF2015 fish datasets, respectively. Lin et al. [23] introduced the RoIMix methodology, which involves factors related to the proximity and occlusion that are typical of interactions between underwater organisms. By simulating target overlap, blending, and blurring from disparate images, the method aims to bolster the interaction representations between images and enhance model generalizability. Jalal et al. [24] proposed the YOLO-Fish detection model, which includes YOLO-Fish-1 and YOLO-Fish-2 iterations. The former iteration refined YOLOv3’s performance by adjusting the upsampling stride, while the latter added a spatial pyramid pooling layer to better capture fish appearances in dynamic settings. Zhang et al. [25] reconceptualized the convolution module, network structure, loss function, and detection head for the YOLOv4 network, incorporating attention mechanisms and activation functions. The resulting algorithm not only achieved a 91.1% detection accuracy but also achieved a clock at 58.1 frames per second (FPS) on the URPC dataset, underscoring its real-time detection capabilities. Addressing the need for lightweight models, Liu et al. [26] devised Tuna-YOLO, optimized for tuna detection and suited for mobile device deployment and real-time applications. Similarly, HU et al. [27] crafted a swift and cost-effective detection system, leveraging underwater imaging coupled with deep learning frameworks to monitor fish behavior within hybrid aquaculture contexts. Moreover, Zhou et al. [28] developed a precise detection method for oriental Takifugu rubripes based on YOLOv7, which is adept at managing multiscale detection of small targets and enhancing information extraction—imperative for smart fish farming. While these studies address various aspects of fish recognition, such as model training speed, detection speed, and feature extraction efficacy, challenges remain. The datasets employed primarily expanded the data volume without ample consideration of the image quality or the morphological details of the fish under diverse environmental conditions, illumination conditions, or viewing angles. Given the three-dimensional nature of fish movement in water, the recognition process necessitates gathering morphological data from multiple perspectives to refine the model’s accuracy and generalizability. Furthermore, intricate underwater settings demand focused detection of minute targets and the discernment of granular features to minimize false positives and negatives. Crucially, an equilibrium between model compactness and detection performance is essential for informing practical applications in aquaculture and production deployment.

In this paper, the SD-YOLOv8 detection model is introduced, utilizing the advanced YOLOv8 network architecture for enhanced detection of *S. dumerili*. Given the limited availability of *S. dumerili* data in existing public datasets, this study undertakes the creation of a comprehensive dataset of *S. dumerili*. The dataset encompasses a variety of angles, lighting conditions, and resolutions. To further refine the dataset, image augmentation techniques are deployed on the blurred and low-light images, bringing the defining features of *S. dumerili* such as color and texture into sharper focus. The YOLOv8-based model architecture is meticulously reworked to improve the detection process. In this renovation, a dedicated layer for small object detection is introduced, along with sophisticated detection heads. Deformable convolutions are integrated into the architecture, enabling the model to fine-tune sampling offsets and weights, and, in turn, reducing the granularity of information loss. Additionally, the model assimilates the BAM attention mechanism and a sophisticated SPPF layer. These modifications facilitate the model’s adaptability to a spectrum of angles and resolutions across both spatial and channel dimensions, which broadens the scope of information fusion and significantly improves the model’s generalizability. To enhance the detection of objects that are overlapping, blurry, or small—especially at the image peripheries, a loss function is proposed. This function is designed to improve the model’s convergence speed and precision. When pitted against other object detection models specializing in *S. dumerili* detection, the SD-YOLOv8 model clearly outperforms the other models in terms of detection performance, as evidenced by comparative evaluations. Such assessments underscore the model’s superior capabilities and confirm the efficacy of the proposed enhancements in real-world applications.

The contributions of this paper are as follows:

(1) Creation of an *S. dumerili* dataset. We simulated real-world scenarios and constructed a dataset with diverse angles, lighting conditions, and resolutions, specifically focusing on *S. dumerili* The dataset was further enhanced using image augmentation techniques to highlight the appearance features of *S. dumerili*.

(2) Redesigned YOLOv8 network architecture. We improved the efficiency of *S. dumerili* detection by introducing new components to the YOLOv8 architecture. This includes adding a small object detection layer and detection heads, incorporating deformable convolutions for better information sampling, and integrating BAM attention and an improved SPPF layer to handle different angles and resolutions.

(3) Inner-MPDIoU loss function. To enhance the model’s ability to detect overlapping, blurry, and small objects at the edges, we propose the Inner-MPDIoU loss function. This loss function improves the convergence speed and accuracy during training, leading to better detection performance.

## 2. Methods

### 2.1. SD-YOLOv8

**Figure 1:**
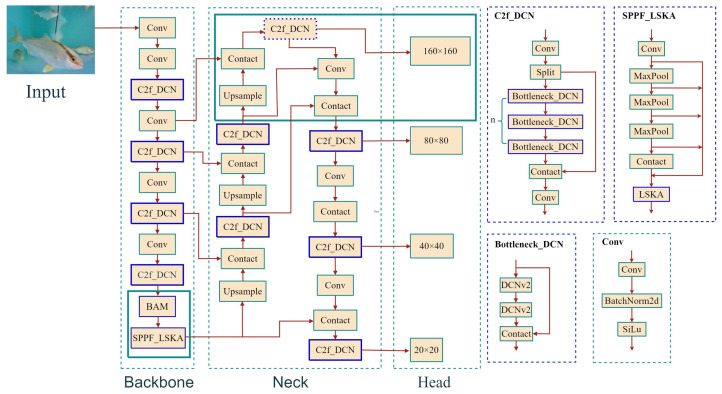
SD-YOLOv8 structure.

The original YOLOv8 model consists of three components: the backbone network, the neck network, and the detection head. The backbone network is responsible for image feature extraction, the neck network handles feature fusion, and the detection head performs object detection at different scales. In this paper, improvements were made to each part of the study to enhance the efficiency of fish detection via SD-YOLOv8, as shown in Figure 1. The backbone utilizes the CSPNet processing concept from YOLOv5, built upon the DarkNet53 feature extraction network to process image features. First, a deformable convolution network (DCNv2) [29] was introduced to the C2f module, allowing for expanded sampling-point offsets and enriched multiscale sampling information, which can effectively cope with the distortion of an underwater environment. DCNv2 incorporates weight coefficients to enhance the accuracy of feature extraction. Second, the bottleneck attention mechanism (BAM) was employed to downsample the mapped image information, creating a multiparameter hierarchical attention structure to enhance channel and spatial mapping capabilities. Finally, a large-kernel separable attention (LSKA) [30] was used to strengthen the robustness of shape information encoding in feature representation, improving the long-range dependency and enhancing the feature fusion capability of the SPPF structure. The neck adopts the PANet concept to further enhance feature fusion at different scales, making it more suitable for object detection. Semantic information extracted from different levels of the backbone network is downsampled and used as input for PANet. Additionally, a small object detection layer was added to the neck to address situations where fish features are not prominent in low-light environments. It concatenates shallow and deep feature maps for detection and adds an auxiliary detection head to the head network for detecting small objects. The detection head adopts a decoupled head method to accelerate model convergence by separating the regression and prediction branches. The regression branch is evaluated using bounding box loss, which includes CIou [31] and distributional focal loss components. The prediction branch is evaluated using binary cross-entropy loss. Both branches are learned separately and then merged. To address the lack of advantages of the complete intersection over union (CIOU) metric in handling overlapping fish bodies, the Inner-MPDIoU metric, a loss function that fully utilizes geometric features of bounding boxes and includes auxiliary bounding boxes, is used to enhance feature convergence and improve model regression efficiency.

### 2.2. Small Object Detection Layer

The original YOLOv8 architecture, which utilizes downsampling layers with larger strides within its neck network, plays a pivotal role in reducing the model’s complexity and streamlining training and inference, contributing to a more lightweight model. However, this configuration poses significant challenges for deep-level feature maps in terms of capturing details about small objects, a drawback that becomes particularly evident in the realm of fish detection. Specifically, small *S. dumerili* that are positioned on the periphery of the image or shrouded within low-light conditions may elude comprehensive detection due to these inherent limitations. The peripheral or low-light presence of these specimens underscores the need for a more nuanced approach that can effectively handle the various challenges presented by the complex, dynamic, and often unpredictable nature of aquatic environments. This paper presents an innovative redesign of the feature extraction mechanism in the neck network to remedy this. The new design incorporates a layer dedicated to small object detection along with an accompanying detection head. The restructured neck network includes supplementary upsampling and convolutional layers that not only deepen the architecture but also widen the receptive field. This enhancement bolsters the integration of features across different levels, leveraging contextual information more effectively and improving the depiction of characteristics specific to *S. dumerili*. Furthermore, the introduction of a specialized detection head for small objects, which employs shallower, higher-resolution feature maps, represents a significant step forward. This head, in combination with the preexisting three heads, culminates in a four-head detection system. This comprehensive structure facilitates the concurrent detection of objects across multiple regions, thereby broadening the scope and speed of detection and curbing the occurrence of missed targets and false positives. This multifaceted approach signifies a substantial advancement in object detection technology, particularly for the accurate identification of small objects such as *S. dumerili* under challenging conditions.

### 2.3. C2f_DCN

**Figure 2:**
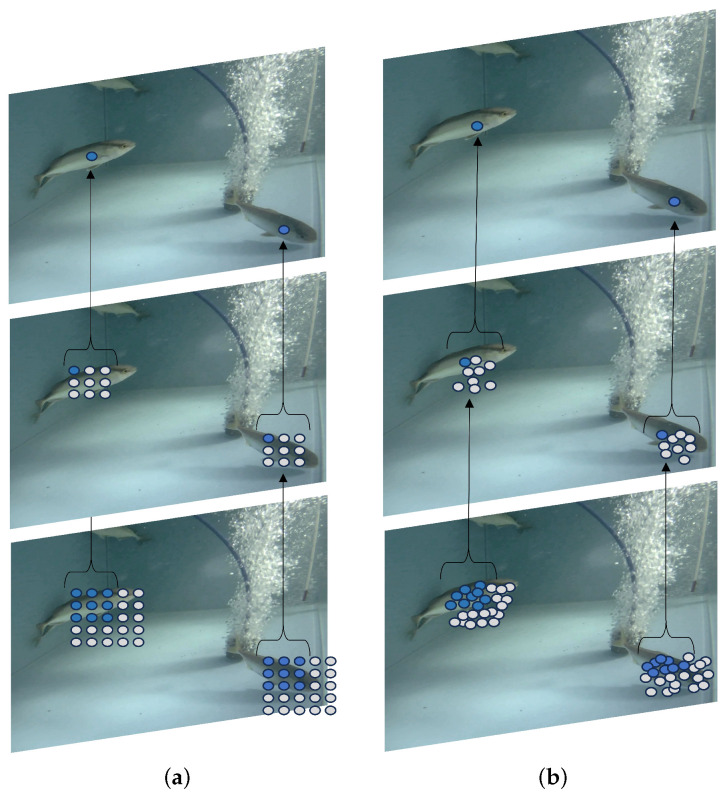
Visualization of standard convolution and deformable convolution. (**a**) Standard convolution with the fixed receptive field. (**b**) Deformable convolution with the adaptive receptive field.

In the YOLOv8 architecture, the C2f module plays a critical role in feature fusion by combining convolutional, splitting, and bottleneck blocks to extract and merge both low-level and high-level features from the input image. This fusion process capitalizes on detailed and semantic information to amalgamate feature maps at varying depths. A limitation of the traditional bottleneck block within the C2f module, as depicted in Figure 2a, is its reliance on fixed 3 × 3 convolutions at predetermined locations on the feature map, which is not optimal for object features that vary in position and scale. This paper introduces an enhancement to the conventional C2f module aimed at overcoming the challenges of scale variation in *S. dumerili*, such as nonlinear aberrations in underwater images, varying distances of the fish from the camera, and incomplete outlines of the fish. The innovation involves the substitution of the fixed 3 × 3 convolution within the bottleneck block with deformable convolution, as described in [32]. This alternative, as visualized in Figure 2b, introduces offset adjustments to standard convolutions, enabling deformable convolution to adapt more effectively to the distinct shapes and sizes of targets. The deformable convolution network v2 (DCNv2) further refines this approach by incorporating additional deformable layers, thus enhancing the convolution kernel’s sampling capacity. Moreover, DCNv2 learns the sampling points’ weight information in tandem with offset learning, which serves to substantially reduce convolutional sampling disruptions caused by extraneous elements. This learned weight information is integral in minimizing the influence of irrelevant factors during the sampling process. The mathematical representation of the weight information is encapsulated in the following equation:

(1) $$y\left(p_{0}\right)=\sum_{p_{n}\in R}\begin{array}{c} \left.w\left(p_{n}\right)\cdot x\left(p_{0}+p_{n}+\Delta p_{n}\right)\cdot \Delta m_{n}\right., \end{array}$$

where $p_{n}$ denotes a predetermined offset, $\Delta p_{n}$ represents the learned offset for the deformable convolution, and $\Delta m_{n}$ is a learnable weight used for end-to-end training. The terms $w\left(p_{n}\right)$, $x\left(p_{0}\right)\left(p_{0}\right)$, and $y\left(p_{n}\right)$ refer to the weights at position $p_{n}$. The pixel feature at position *x* is obtained from the input feature map, and the image feature at position $p_{0}$ is obtained from the output feature map.

### 2.4. Bottleneck Attention Module

**Figure 3:**
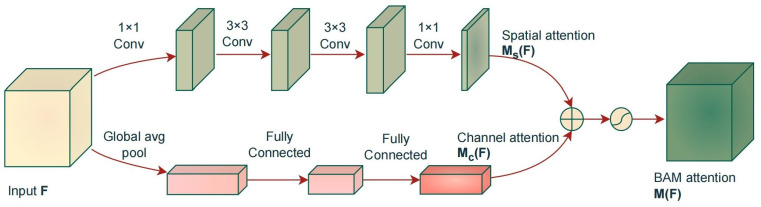
The structure of BAM.

This paper introduces the BAM to enhance the model’s focus on *S. dumerili* features to mitigate interference from underwater bubbles, turbid water quality, and feed obstruction. The BAM decomposes the input image into spatial attention and channel attention components. The spatial attention component focuses on learning the content information of the image, while the channel attention component focuses on learning the positional information of the image. The structure of the BAM is shown in Figure 3. Spatial attention utilizes dilated convolutions to emphasize or suppress features at different positions, thereby expanding the receptive field and enhancing the ability to utilize contextual information, thus strengthening the spatial mapping capability. Channel attention adaptively adjusts the feature response of each channel by leveraging the relationships between channel branches. The calculation formula for the BAM is shown as follows:

(2) $$M\left(F\right)=M_{s}\left(F\right)+M_{c}\left(F\right),$$

where *F* represents the input feature map. $M_{s}\left(F\right)$ refers to the spatial attention processing result, $M_{c}\left(F\right)$ denotes the channel attention mechanism processing result, and $M\left(F\right)$ represents the overall BAM attention.

### 2.5. SPPF_LSKA

YOLOv8 utilizes the fast spatial pyramid pooling (SPPF) technique, which applies a series of max pooling layers to different scale feature maps to generate fixed-sized vector outputs, addressing the issue of information distortion caused by traditional pooling methods. In traditional YOLOv8, the SPPF layer incorporates a 1×1 convolution for information fusion, achieving satisfactory results in tasks with a single background. However, this approach fails to balance the structural and channel adaptability of image information when dealing with complex backgrounds. Therefore, redesigning this component is of high importance for improving fish recognition capabilities.

**Figure 4:**
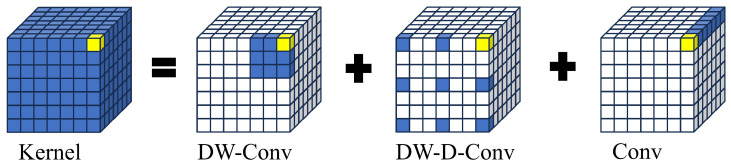
Illustration of large-kernel convolution. The colored grids represent the positions of the convolution kernels, while the yellow grids represent the central points of the grids. The process involves decomposing a 13×13 convolution into a 5×5 depthwise convolution, a 5 × 5 depthwise dilated convolution, and a 1×1 convolution.

**Figure 5:**
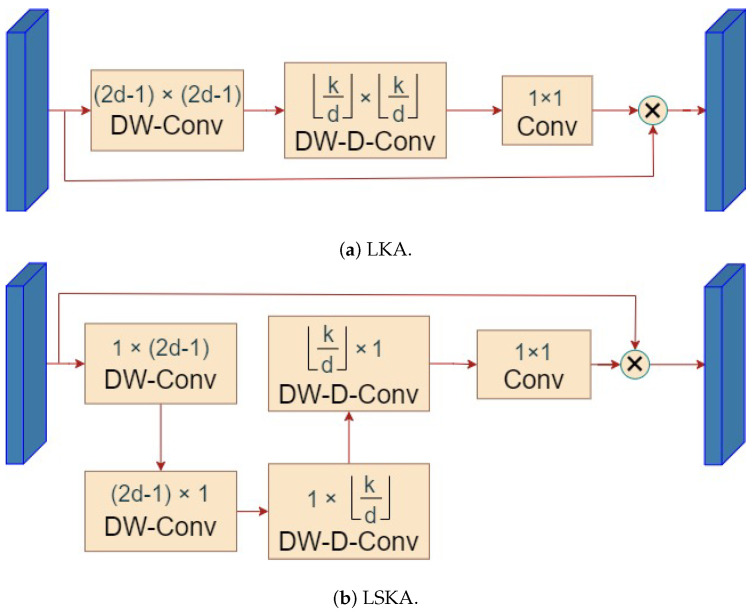
Visualization of kernel attention. (**a**) The LKA structures, and (**b**) the LSKA structures.

Large separable kernel attention (LSKA) is introduced for reconstructing the SPPF layer, which is tailored specifically for vision tasks. A large-kernel convolution captures a wider range of contextual information, aiding the model in better learning object positions and relationships in the image. However, simply enlarging the convolution kernel leads to increased parameters and computational complexity, which is detrimental to model training. In light of this, large-kernel attention (LKA) decomposes large convolution kernels into a spatial local convolution (depthwise convolution), a spatial long-range convolution (depthwise dilated convolution), and a channel convolution. Specifically, the K×K convolution is reconfigured into a depthwise dilated convolution with a dilation factor of *d*, which results in a spatial kernel size of $\left\lfloor \frac{K}{d}\right\rfloor \times \left\lfloor \frac{K}{d}\right\rfloor$. This is followed by a depthwise convolution with a kernel size of $\left(2d-1\right)\times \left(2d-1\right)$, and subsequently, Sa 1×1 convolution is applied [33]. This decomposition strategy reduces the computational and parameter burdens associated with purely enlarging the convolution kernel. The convolution decomposition method is illustrated in Figure 4, and the LKA structure is shown in Figure 5a. The calculation of the LKA is shown as follows:

(3) $$\overset{-}{Z^{c}}=\sum_{h,w}W_{\left(2d-1\right)\times \left(2d-1\right)}^{c}*F^{c},$$

(4) $$Z^{C}=\sum_{H,W}W_{\left\lfloor \frac{k}{d}\right\rfloor \times \left\lfloor \frac{k}{d}\right\rfloor }^{C}*{\overset{-}{Z}}^{C},$$

(5) $$A^{C}=W_{1\times 1}*Z^{C},$$

(6) $${\overset{¯}{F}}^{C}=A^{C}⊗F^{C},$$

where $F\epsilon R^{c\times h\times w}$ represents the input feature map, *c* is the number of input channels, and *h* and *w* denote the height and width of the feature map, respectively. *d* represents the dilation rate, $W^{c}$ represents the number of channels in the convolutional kernels, $F^{c}$ represents the number of channels in the feature map, and $\;{\bar{Z}}^{c}$ represents the output of the depthwise convolution with the input feature map *F* using a kernel of size $\left(2d-1\right)\times \left(2d-1\right)$. This depthwise convolution captures local spatial information and compensates for the subsequent depth expansion convolution $Z^{C}$. *k* represents the kernel size, and $Z^{C}$ is the output of the depth expansion convolution obtained by convolving ${\bar{Z}}^{c}$ with a kernel of size $\frac{k}{d}$ × $\frac{k}{d}$. The $\left\lfloor .\right\rfloor$ denotes the floor operation. $A^{C}$ represents the attention map obtained by convolving the depth expansion convolution with a 1×1 kernel. The output ${\bar{F}}^{C}$ of the LKA is the Hadamard product (denoted by ⊗ ) of the attention map $A^{C}S$ and the input feature map $F^{C}$.

To further reduce computational complexity, LSKA splits the depthwise convolution layer and depth expansion convolution into two cascaded one-dimensional separable convolutions while preserving larger convolution kernels; the LSKA structure is shown in Figure 5b. The calculations for LSKA are shown as follows:

(7) $$\overset{-}{Z^{c}}=\sum_{h,w}W_{\left(2d-1\right)\times 1}^{c}*\left(\sum_{h,w}W_{1\times \left(2d-1\right)}^{c}*F^{C}\right),$$

(8) $$Z^{C}=\sum_{h,w}W_{\left\lfloor \frac{k}{d}\right\rfloor \times 1}^{c}*\left(\sum_{h,w}W_{1\times \left\lfloor \frac{k}{d}\right\rfloor }^{c}*{\overset{-}{Z}}^{c}\right),$$

(9) $$A^{C}=W_{1\times 1}*Z^{C},$$

(10) $${\overset{¯}{F}}^{C}=A^{C}⊗F^{C}.$$

### 2.6. Inner-MDPIoU

The YOLOv8 loss function consists of two parts: classification loss and regression loss. The classification loss is calculated using binary cross-entropy loss, while the regression loss is calculated using a combination of distribution focal loss [34] and bounding box regression (BBR) [35]. However, since this study focuses only on classifying *S. dumerili*, which has only one category, the binary cross-entropy loss for that category is equal to 0. Therefore, the final loss function can be represented as follows:

(11) $$\;\;\;f_{loss}=\lambda_{1}f_{DFL}+\lambda_{2}f_{BBR},$$

The distribution focal loss (DFL) optimizes the probabilities of the left and right positions closest to the label *y* in the form of cross-entropy. This helps the network quickly focus on the distribution of the target position and its neighboring regions. It can be represented as follows:

(12) $$f_{DFL}\left(S_{i}{\;,\;S}_{i+1}\right)=-\left(\left(y_{i+1}-y\right)\log\left(S_{i}\right)+\left(y-y_{i}\right)log\left(S_{i+1}\right)\right),$$

where $y_{i\;}$ and $y_{i+1}$ represent the values approaching the continuous label *y* from the left and right sides, respectively, satisfying $y_{i}<y<y_{i+1}$. Additionally, the equation $y=\Sigma_{1=0}^{n}P\left(y_{i}\right)y_{i\;}$ describes the relationship between *y* and the probabilities $P\left(y_{i}\right)$, where $P\left(y_{i}\right)$ can be implemented using a softmax layer denoted as $S_{i}$ in the formula.

**Figure 6:**
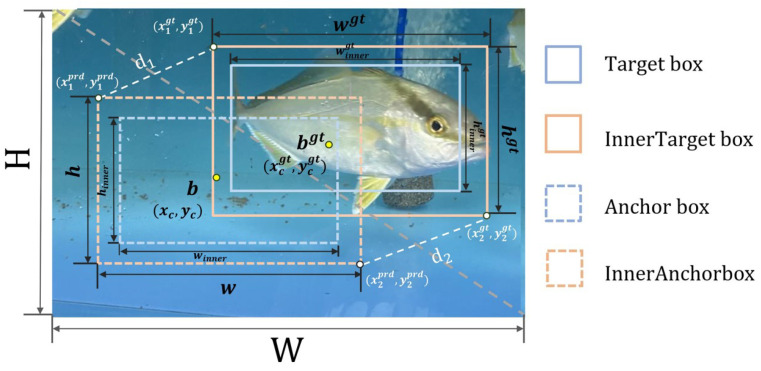
Calculation diagram of the Inner-MPDIou model.

During the data collection process, healthy fish were observed swimming actively in the water, and there was overlap and occlusion among the fish groups. Therefore, a loss function that can handle targets in a refined manner and robustly handle folding is needed. This study optimized the BBR loss function by using a new similarity metric called the Inner-MPDIoU metric to compute the IoU loss function by incorporating auxiliary bounding boxes. The Inner-MPDIoU calculation and parameters are shown in Figure 6. In the Inner-MPDIoU calculation, the MPDIoU component leverages the geometric properties of BBR to minimize the points distance $d_{1}$ and $d_{2}$ between the top-left and bottom-right corners of the predicted box and the ground-truth box. This measures the similarity between the predicted and ground-truth boxes, which can be adapted to overlapping or nonoverlapping bounding box regression. In the training phase, each bounding box $b=[x_{c},y_{c},w_{inner},h_{inner}]$ predicted by the model is forced to approach its ground-truth box $b^{gt}=[x_{c}^{prd},y_{c}^{prd},w_{inner}^{gt},h_{inner}^{gt}]$ by minimizing the loss function of the two points [36]. The Inner-IoU component adjusts the size of the auxiliary bounding boxes relative to the actual box using the scale ratio, which can control the scale size of the auxiliary bounding boxes [37]. Since the detection target in this experiment was fish, more detailed image information was needed. Therefore, the ratio was set to 1, meaning that the auxiliary box is smaller than the actual box. When the auxiliary box was smaller than the actual box, the effective range of the prediction was smaller than the IoU loss. However, the gradient magnitude obtained from the auxiliary box was greater than that from the IoU loss, accelerating the convergence of high IoU samples and obtaining more detailed image information by reducing the size of the ground-truth box. The combination of these two components robustly improved regression accuracy and convergence speed. The Inner-MPDIoU calculation formula can be represented as follows:

(13) $$d_{1}^{2}={\left(x_{1}^{prd}-x_{1}^{gt}\right)}^{2}+{\left(y_{1}^{prd}-y_{1}^{gt}\right)}^{2},$$

(14) $$d_{2}^{2}={\left(x_{2}^{prd}-x_{2}^{gt}\right)}^{2}+{\left(y_{2}^{prd}-y_{2}^{gt}\right)}^{2},$$

(15) $$IoU=\frac{\;Target\;box\;\cap \;Ground\;truth\;box}{Target\;box\;\cup Ground\;truth\;box},$$

(16) $$MPDIoU=IoU-\frac{d_{1}^{2}}{W^{2}+H^{2}}-\frac{d_{2}^{2}}{W^{2}+H^{2}},$$

(17) $$L_{MPDIoU}=1-MPDIoU,$$

where $d_{1}$ represents the distance between the top-left corners $\left(x_{1}^{prd},y_{1}prd\right)$ and $\left(x_{1}^{gt},y1_{g}t\right)$ of the predicted bounding box and the ground-truth bounding box, respectively, while $d_{2}$ represents the distance between the top-right corners $\left(x_{2}^{prd},y_{2}^{p}rd\right)$ and $\left(x_{2}^{gt},y2gt\right)$. W and *H* represent the width and height of the input image, respectively.

(18) $$b_{l}^{gt}=x_{c}^{gt}-\frac{w^{gt}*ratio}{2},b_{r}^{gt}=x_{c}^{gt}+\frac{w^{gt}*ratio}{2},$$

where $B^{gt}$ and *B* represent the ground-truth bounding box and anchor box, respectively. $\left(x_{c}^{gt},y_{c}^{gt}\right)$ represents the coordinates of the center point of the ground-truth box, while $\left(x_{c},y_{c}\right)$ represents the center point of the anchor box and the inner anchor box. $W^{gt}$ and $h^{gt}$ represent the width and height of the ground-truth box, respectively.

(19) $$b_{t}^{gt}=y_{c}^{gt}-\frac{h^{gt}*ratio}{2},b_{b}^{gt}=y_{c}^{gt}+\frac{h^{gt}*ratio}{2},$$

(20) $$b_{l}=x_{c}-\frac{w*ratio}{2},b_{r}=x_{c}+\frac{w*ratio}{2},$$

where *w* and *h* represent the height and width of the anchor box, respectively.

(21) $$b_{t}=y_{c}-\frac{h*ratio}{2},b_{b}=y_{c}+\frac{h*ratio}{2},$$

(22) $$inter=\left(\min\left(b_{r}^{gt},b_{r}\right)-\max\left(b_{l}^{gt},b_{l}\right)\right)*\left(\min\left(b_{b}^{gt},b_{b}\right)-\max\left(b_{t}^{gt},b_{t}\right)\right)$$

(23) $$union=\left(w^{gt}*h^{gt}\right)*{\left(ratio\right)}^{2}+\left(w*h\right)*{\left(ratio\right)}^{2}-inter,$$

(24) $${IoU}^{inner}=\frac{inner}{union},$$

(25) $$L_{Inner-MPDIou}=L_{MPDIoU}+IoU-{IoU}^{inner}.$$

## 3. Materials

### 3.1. Experimental Environment and Parameter Settings

All experiments in this paper were conducted on the same computer with the following specifications: Windows 10 operating system, Intel^®^ Xeon^®^ Silver 4100 CPU, and NVIDIA GeForce RTX 2080 Ti GPU. PyTorch version 2.0.0 and CUDA version 11.7 were used. The experiments were conducted for 100 epochs with a batch size of 16 and a learning rate of 0.01 to evaluate the model performance. The results were optimized using the SGD optimizer.

### 3.2. Dataset

In order to evaluate the detection robustness of the SD-YOLOv8 model, we conducted experiments on two datasets: the *S. dumerili* dataset and a real-world dataset. Firstly, we trained the SD-YOLOv8 model using the *S. dumerili* dataset. This dataset provided a foundation for the model’s initial training and performance assessment. To further validate the model’s robustness, we constructed a separate dataset by gathering images from FishBase (https://www.fishbase.se, accessed on 21 April 2024) and the Aquarium dataset (https://public.roboflow.com/object-detection, accessed on 21 April 2024). The real-world dataset encompasses a broader spectrum of environmental conditions and object variations, thereby furnishing us with the means to rigorously evaluate the model’s detection proficiencies across a plethora of settings. Crucially, this dataset enables an effective validation of SD-YOLOv8’s capacity to discern and categorize entities beyond the realm of *S. dumerili* biology. By integrating this supplementary dataset, our objective is to augment the model’s aptitude for precise detection and classification of objects, transcending the limitations imposed by the initial training dataset. In essence, the inclusion of a diverse real-world dataset serves to fortify the robustness and versatility of the SD-YOLOv8 model. It allows for the assessment of the model’s performance in scenarios that deviate from the controlled conditions of the original training environment. This is pivotal for ensuring that the model can generalize its learning to novel situations, thereby enhancing its applicability and reliability in practical, real-world applications.

#### 3.2.1. *S. dumerili* Dataset

**Figure 7:**
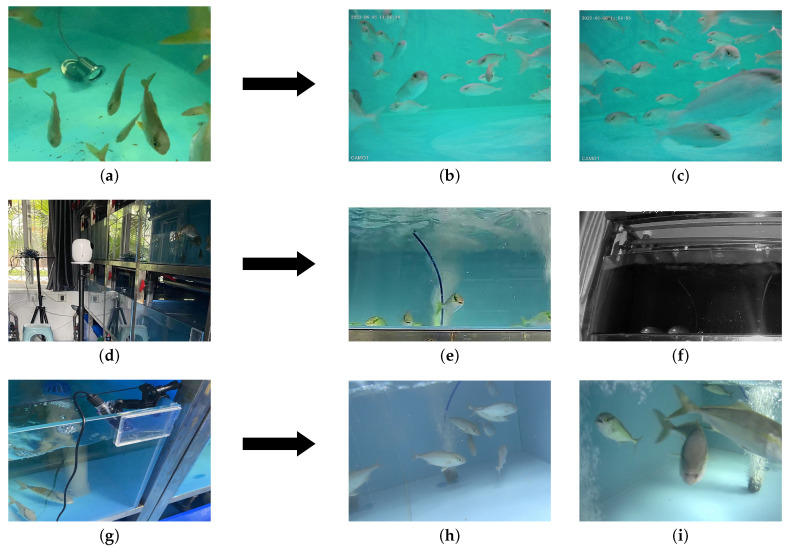
Images with different shooting angles. (Images (**a**,**d**,**g**) were taken by a Barlus underwater camera, a TP-Link network camera, and an SJCAM action camera, respectively. Images (**b**,**c**,**e**,**f**,**h**,**i**) are the captured images from the aforementioned cameras).

This paper compiled a dataset of *S. dumerili* images under laboratory conditions with variances in angles, lighting, and resolutions. Imaging data for *S. dumerili* were acquired from the Biological Breeding Center at the Southern Marine Science and Engineering Laboratory (ZHANJIANG) within recirculating aquaculture systems. These images were captured both above and beneath the water surface, under various lighting conditions spanning the early morning to the evening of a day. The dataset includes 1071 images, and it was segmented into training, validation, and testing subsets in a 7:2:1 ratio. Table 1 details the resolution of the capturing equipment, and Figure 7 shows the experimental capture angles and image collection methodology.

Proper preprocessing of underwater datasets is crucial for correcting image color and enhancing image quality and clarity, which are essential for subsequent model training and prediction. Effective preprocessing steps ensure that the inherent distortions and variations in underwater images are mitigated, thereby providing a more reliable and accurate input for deep learning models [38]. Among the training set, there were 460 original images directly extracted from the captured videos and 289 images from applied augmentation software. Of the 289 images, there were 156 images with diverse lighting conditions and 133 images with blurry boundaries; we employed image augmentation software [39] to augment the training of these images. Among the augmentation methods used, we applied the sigmoid contrast technique [40] to enhance the contrast of low-light images, which rectified the images’ color performance, improving their visibility and ensuring accurate object detection. Additionally, we utilized the canny method [41] to emphasize blurry boundaries, making them more distinct and aiding in the model’s ability to accurately detect and delineate objects with unclear edges. These augmented images played a crucial role in training the SD-YOLOv8 model to handle a wide range of lighting conditions and object boundaries, ultimately enhancing its generalization capabilities. However, for the validation and testing sets, no such augmentation was enacted, as their role was to gauge the model’s ability with real-world applications.

To optimize the cost of manual labeling, we adopted a two-step approach for labeling our dataset. Initially, we leveraged the LabelImg [42] tool to manually label 200 images. These labeled images served as a foundation for training our model to detect and label the remaining images in an automated manner. Using the pretraining model, we applied automated labeling to the remaining images. Although this approach may introduce some inaccuracies in the initial labels, we subsequently conducted a thorough review of all labels. During this review process, we meticulously checked and modified any inaccurate labels, ensuring the highest level of accuracy and precision in the dataset. By combining manual labeling with automated labeling using the pretraining model, we were able to significantly reduce the amount of human effort and resources required for the dataset labeling process. This approach effectively balanced the need for accurate labeling while optimizing the time and manpower involved in the overall process.

#### 3.2.2. Real-World Dataset

The real-world dataset was collected from FishBase and the Aquarium dataset. FishBase is an open-source fish database created and maintained by the Leibniz Institute of Oceanology, which provides researchers with comprehensive data on species, regional distribution, and population density [43]. We took the species of *S. dumerili* in our constructed dataset, which contained 96 wild *S. dumerili* images. The Aquarium dataset was collected by Roboflow from two aquariums in the United States, The Henry Doorly Zoo in Omaha (16 October 2020) and the National Aquarium in Baltimore (14 November 2020) [44], and is composed of 638 images that cover different classes of the following marine life: fish, jellyfish, penguins, sharks, puffins, stingrays, and starfish. The entire real-world dataset contains 735 images and covers eight classes, and it was split into a training set and a test set in an 8:2 ratio.

### 3.3. Evaluation Metrics

To evaluate the algorithm model in the experiments, performance metrics based on neural network models [45] are used in this paper. The precision, recall, F1 score, and average precision are adopted as evaluation metrics.

Precision represents the ratio of true-positive results to the total number of predicted samples. The formula is as follows:

(26) $$Precision=\frac{TP}{TP+FP},$$

where TP represents the number of actual *S. dumerili* samples correctly identified as *S. dumerili*, and FP represents the number of non-*S. dumerili* samples incorrectly identified as *S. dumerili*.

Recall represents the proportion of correctly identified samples to the total samples in the dataset. The formula is as follows:

(27) $$Recall=\frac{TP}{TP+FN},$$

where FN represents the number of actual *S. dumerili* incorrectly identified as *S. dumerili*. The F1 score is the weighted average of precision and recall and is a comprehensive metric for evaluating the accuracy and recall of a model in detection tasks. The F1 score ranges from 0 to 1, and a higher value indicates better model performance. When both the precision and recall are high, the F1 score is also high, indicating that the model performs well in the detection task. The formula is defined as follows:

(28) $$F1=\frac{2}{{Recall}^{-1}+\;{Precision}^{-1}}=2\cdot \frac{Precision\cdot Recall}{Precision+Recall},$$

In the context of *S. dumerili* detection, the average precision (AP) is calculated by taking the average precision at various recall levels, providing an overall assessment of the precision–recall trade-off. The mean average precision (mAP) represents the average AP for different categories. In this specific paper, as only *S. dumerili* is detected, the AP is equal to the mAP. The formulas for the AP and mAP are defined as follows:

(29) $$AP=\int_{0}^{1}p(R)dR,$$

(30) $$mAP=\frac{\sum_{1}^{N}\int_{0}^{1}P(R)dR}{N},$$

where N represents the number of classes. When there is only one class, the AP is equal to the mAP.

## 4. Results and Analysis

### 4.1. Model Comparison

**Figure 8:**
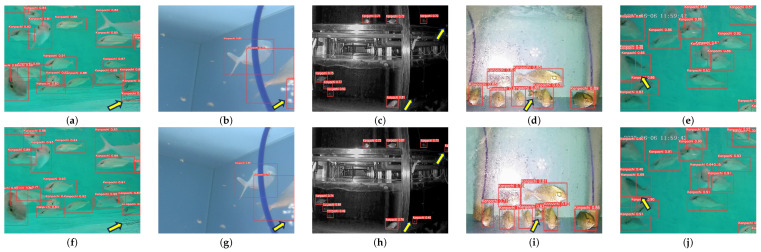
Comparison of the original network and improved results.The original YOLOv8n detection performance as (**a**–**e**) shown. SD-YOLOv8n detecion performance as (**f**–**j**) shown.

Different *S. dumerili* images with different backgrounds are selected to compare the improvement of the algorithm.The model detection comparison is shown in Figure 8. Figure 8 provides an illustrative comparison between the original YOLOv8n detection results (panels a, b, c, d, and e) and the proposed SD-YOLO method presented in our paper (panels f, g, h, i, and j). Specifically, the pairs of (a) and (f) and of (b) and (j) depict cases of missed detections, such as baits and splash bubbles, (c) and (h) show missed detection as well night detection performance, (d) and (i) demonstrate missed detections of small fishes, and (e) and (j) highlight fish body overlap. Due to the redesigned feature extraction and fusion blocks in the network, the model’s ability to learn and generalize *S. dumerili* details is enhanced. The comparison images show that the proposed method improves the recognition ability of *S. dumerili* for complex backgrounds, especially for complex backgrounds and overlapping or occluded fish bodies.

**Figure 9:**
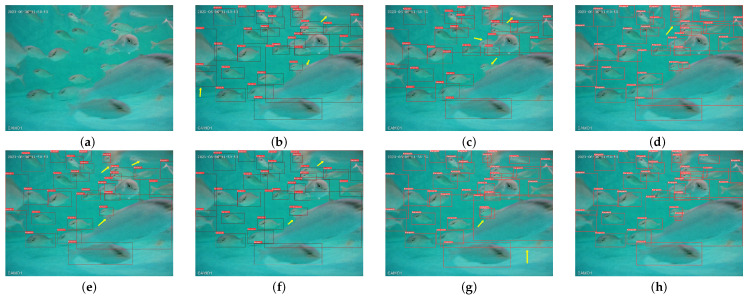
Comparison results of different detection model: (**a**) original picture; (**b**) SSD; (**c**) YOLOv4-tiny; (**d**) YOLOv5s; (**e**) YOLOX-s; (**f**) YOLOv7; (**g**) YOLOv8n; (**h**) SD-YOLOv8.

We conducted a comparative analysis of our proposed model against mainstream models, employing the S. dumerili annotated dataset as input for the Faster-RCNN [46], RetinaNet [47], SSD [48], YOLOv4-tiny, YOLOv5-s, YOLOX-s [49], YOLOv7, and YOLOv8n models. Our evaluation encompassed precision, recall, F1 score, ${\mathrm{mAP}}_{@0.5}$, parameter count, computational complexity, and model size as performance metrics. Upon comparing our two-stage object detection model, SD-YOLOv8, with Faster R-CNN, CenterNet, and RetinaNet, it becomes apparent that SD-YOLOv8 surpasses the two-stage network in all metrics. In contrast to the one-stage object detection model, SSD, and other YOLO series models, our innovative model demonstrates notable improvements in precision. Specifically, it outperforms SSD by 8.9%, YOLOv4-tiny by 9.6%, YOLOv5 by 2.7%, YOLOX-s by 3.1%, YOLOv7 by 4.8%, and YOLOv8n by 4.1%. Moreover, it exhibits an 8.7%, 11.5%, 2.5%, 1.3%, 3.3%, and 3.5% increase in ${\mathrm{mAP}}_{@0.5}$ compared to SSD, YOLOv4-tiny, YOLOv5, YOLOX-s, YOLOv7, and YOLOv8n, respectively. Furthermore, SD-YOLOv8n showcases the highest F1 score among both one-stage and two-stage models. In terms of model size, our proposed model is comparable to YOLOv5 and 1.6MB smaller than YOLOv8n, while significantly outperforming other models listed in Table 2 in terms of compactness. Additionally, Figure 9 shows the detection results of our experimentally improved model, which achieved a 4.1% improvement in accuracy compared to the YOLOv8n baseline model. It also yields higher values of precision, recall, F1 score, and ${\mathrm{mAP}}_{@0.5}$ than SSD, YOLOv4-tiny, YOLOv5, YOLOX-s, and YOLOv7. In terms of parameter count, computational complexity, and model size, our proposed model competes with mainstream models.

**Figure 10:**
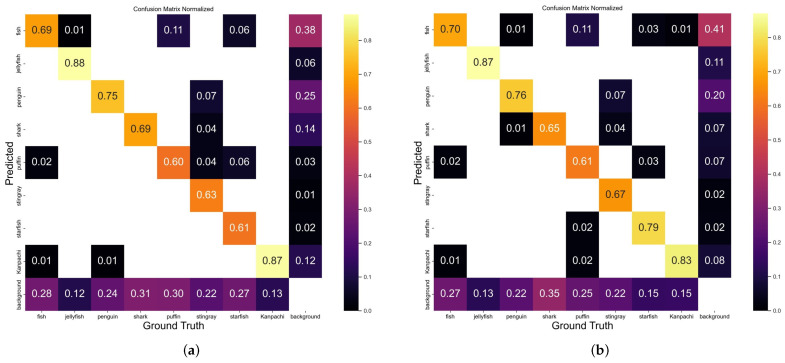
Confusion matrices of SD-YOLOv8 and YOLOv8n on *S. dumerili* dataset and real-world dataset. SD-YOLOv8 (**a**) exhibits superior performance across six classes: fish, penguin, shark, puffin, starfish, and Kanpachi. YOLOv8n (**b**) demonstrates notable capabilities in distinguishing jellyfish and stingrays.

In order to further assess the performance of our model, we conducted experiments on a real-world dataset. The results of the confusion matrix are depicted in Figure 10. Upon examining Figure 10, it becomes evident that SD-YOLOv8 outperforms YOLOv8n in six classes, namely, fish, penguin, shark, puffin, starfish, and Kanpachi. On the other hand, YOLOv8n demonstrates superior capabilities in distinguishing jellyfish and stingrays. However, it is important to note that both models encounter challenges in accurately identifying multiple classes, resulting in occasional misclassifications into unrelated categories.

**Figure 11:**
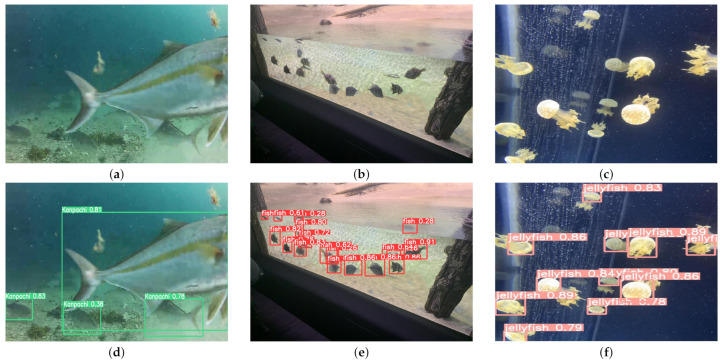
Detection results of SD-YOLOv8 on the real-world dataset.Image (**a**–**c**) are the original images, while images (**d**–**f**) display the SD-YOLOv8 detection results. Among these results, (**d**) demonstrates the capability in detecting wild *S. dumerili*, (**e**) showcases the ability to identify objects across varying scales with a notable emphasis on the detection of diminutive objects, and (**f**) illustrates the model’s resilience against environmental perturbations such as light reflections while maintaining its targeting accuracy.

On the real-world dataset, as illustrated in Table 2, our SD-YOLOv8 model achieves the highest precision of 81.6%, with YOLOX-s closely following at a mere 0.1% lower. However, when it comes to the recall metric, Faster RCNN outperforms all other models with a recall rate of 75.4%. CenterNet, SD-YOLOv8, YOLOv5, YOLOv8n, RetinaNet, SSD, and YOLOv4-tiny exhibit comparable recall metrics, all hovering around 65%. YOLOX-s, on the other hand, demonstrates a lower recall rate. In terms of F1 score, SD-YOLOv8, YOLOv8n, and Faster RCNN showcase the top performances, reaching 73.1%, 71.5%, and 70.6%, respectively. In the ${\mathrm{mAP}}_{@0.5}$ metric, CenterNet outperforms other models by achieving a detection accuracy improvement of 4.7% over Faster RCNN, 19.5% over RetinaNet, 4.4% over SSD, 9.8% over YOLOv4-tiny, 1.2% over YOLOv5, 6.4% over YOLOX-s, 12.1% over YOLOv7, 6.7% over YOLOv8n, and 2.3% over SD-YOLOv8. When considering the parameters metric, YOLOv8n stands out as the most lightweight model, with only 3.1 M parameters. SD-YOLOv8 remains the second smallest model among the compared detection models. In terms of floating point operations per second (FLOPs), the larger-scale detection model Faster RCNN requires 370.2 G FLOPs, similar to its performance on the *S. dumerili* dataset. YOLOv5 and YOLOv4-tiny have computational costs of 6.1 G and 6.5 G FLOPs, respectively, which are still considered low on real-world datasets. With regard to model size, both our proposed SD-YOLOv8 and YOLOv8n exhibit the smallest sizes, weighing in at a mere 7.6 MB. Overall, when compared to different object detection models, SD-YOLOv8 continues to outperform other models on the real-world dataset. Figure 11 presents the adeptness of SD-YOLOv8 in discerning relevant subjects within a real-world dataset. The visuals collectively corroborate the model’s proficiency in reliably distinguishing and excluding non-*S. dumerili* marine entities. For instance, panels (b) and (e) elucidate the model’s capabilities in identifying objects across varying scales with a notable emphasis on the detection of diminutive objects. Meanwhile, panels (c) and (f) exemplify the model’s resilience against environmental perturbations such as light reflections while maintaining its targeting accuracy. These instances are demonstrative of SD-YOLOv8’s formidable object detection competencies in complex, real-world scenarios.

### 4.2. Ablation Experiments

In terms of modules, this study conducted nine ablation experiments to test their performance on the validation set in terms of precision, recall, F1 score, ${\mathrm{mAP}}_{@0.5}$, and ${\mathrm{mAP}}_{@0.5:0.95}$. As shown in Table 3, where YOLOv8n serves as the baseline model, “structure” represents the performance of the model after modifying the network structure. Subsequent experiments gradually added DCNv2, the BAM attention mechanism, the SPPF_LSKA module, and combinations of the two modules to the modified network to evaluate the performance of the models. The improved model achieved increases in accuracy, F1 score, ${\mathrm{mAP}}_{@0.5}$, and ${\mathrm{mAP}}_{@0.5:0.95}$. Specifically, as the accuracy increased by 4.1 percentage points, the F1 score increased by 2.1 percentage points, the ${\mathrm{mAP}}_{@0.5}$ increased by 3.2 percentage points, and the ${\mathrm{mAP}}_{@0.5:0.95}$ increased by 3.2 percentage points.

Upon conducting a more nuanced analysis, it becomes evident that the integration of various modules yielded substantial enhancements to the baseline model. While the C2f_DCN and SPPF_LSKA modules demonstrated remarkable contributions, it is important to acknowledge that other improvement options should not be overlooked. The C2f_DCN module showcased considerable potential in augmenting recall, ${\mathrm{mAP}}_{@0.5}$, and ${\mathrm{mAP}}_{@0.5:0.95}$ metrics. This can be primarily attributed to the utilization of deformable convolutions, which empower the model to selectively capture comprehensive object features within the input images. By adopting this approach, the C2f_DCN module successfully surpasses the limitations of traditional convolutional methods, ultimately leading to improved performance across multiple evaluation criteria. Conversely, the SPPF_LSKA module demonstrated its prowess in precision and the F1 score metrics. By incorporating downsampling layers with larger strides, this module excels in capturing intricate details with finesse, thereby refining the model’s ability to discern objects with exceptional accuracy. The larger strides play a pivotal role in expanding the receptive field of the network, facilitating a more holistic understanding of the underlying features and enabling precise object localization. Although the C2f_DCN and SPPF_LSKA modules made significant contributions to the performance of SD-YOLOv8, it is essential to recognize that the overall improvements are not solely attributed to these modules. Other enhancement options should not be disregarded, as they may have also played vital roles in refining the model’s capabilities. The intricate interplay of various modules, along with diligent fine-tuning and optimization, collectively contribute to the notable performance advancements witnessed in SD-YOLOv8.

In the loss function section, six sets of experiments were designed to assess the impact of various loss functions on model performance. The traditional intersection over union (IoU) algorithm lacks sensitivity to the proximity of overlapping bounding boxes, which complicates the calculation of loss and the execution of gradient backpropagation. The generalized intersection over union (GIoU) [50] introduces a minimum enclosing box as a penalty term, enhancing the gradient optimization of the traditional IoU. However, the convergence with the GIoU is slower, indicating potential for progress. Consequently, the CIoU refines the penalty by incorporating aspects such as bounding box overlap, center distance, and aspect ratio, which facilitates faster convergence and elevates detection efficiency. Nevertheless, the CIoU can be further optimized, particularly by refining aspect ratio differences under comparable conditions. The efficient intersection over union (EIoU) builds on the CIoU by dissecting the factors affecting the aspect ratio, refining the calculation of the aspect ratio loss, and rectifying the imbalance of challenging samples. The scale-IoU (SIoU) metric [51] is a novel approach for loss computations that incorporates the angle, distance, and shape between the actual and predicted boxes, notably for detecting small objects, and significantly improves the resolution of orientation mismatches. MPDIoU, a regression loss function grounded on minimum point distance, addresses the issue of disparate values with identical aspect ratios between the actual and predicted boxes by measuring the distance along two aligned axes, advancing model performance in overlapping scenarios. Despite these advancements, most IoU-based methods have focused on innovating new loss functions to quicken convergence and enhance model proficiency, often neglecting the intrinsic characteristics of the IoU. In response, the inner-IoU metric is introduced, which refines the scaling of anchor boxes by implementing a scale factor, thus bolstering the model’s adaptability across various detectors and detection tasks. This research integrates the Inner-IoU metric, which concentrates on anchor boxes, with the MPDIoU metric, which refines the loss term for boundary boxes. The combined Inner-MPDIoU loss function for bounding boxes significantly improves the efficiency of *S. dumerili* detection. As detailed in Table 4, the Inner-MPDIoU achieves substantial enhancements in metrics such as precision, recall, and F1 score.

## 5. Conclusions

This study created an *S. dumerili* dataset with multiple angles, multiple light sources, and multiple resolutions, providing data support for accurate recognition of *S. dumerili*. The SD-YOLOv8 model was improved by modifying the network structure and adding a small object detection layer and detection head to enhance the efficiency of identifying small fish objects. The DCNv2 fusion with C2f with deformable convolution was introduced into the backbone network to enhance the model’s ability to handle complex information and improve its resistance to interference, while also improving the extraction of fine-scale fish body features. The bottleneck attention module (BAM) was introduced to focus on both spatial and channel information, further enhancing the ability to acquire information on *S. dumerili*. The large separable kernel attention (LSKA) algorithm was used to fuse the spatial pyramid pooling fast (SPPF) algorithm for multiscale feature fusion in the backbone network. Finally, the Inner-MPDIoU metric was used to achieve rapid convergence and regression of the bounding box loss function. The experimental results showed that the improved YOLOv8n model achieved an increase in accuracy from 89.2% to 93.3% and an increase in average detection accuracy from 92.2% to 95.7%, corresponding to increases of 4.1 points and 3.5 points, respectively. The real-world dataset further validated the performance in scenarios that deviate from the controlled conditions of the original training environment.

This study preliminarily established a recognition model for *S. dumerili* in complex environments. In future research, we plan to collect more images under varied environmental conditions and implement tailored preprocessing methods to enhance the dataset’s robustness and applicability. Furthermore, adding more detection of other underwater objects also has great significance in enhancing the model’s comprehensive ability.

## Figures and Tables

**Table 1:** Shooting device information.

Device Name	Resolution		
Barlus Underwater Camera	5 MP	2592 × 1944	25 FPS
IPC5MPW-PBX10	4 MP	2560 × 1440	25 FPS
TP-Link Network Camera	4 MP	2560 × 1440	15 FPS
TL-IPC44B			
SJCAM Action Camera C300	4 K	3840 × 2160	30 FPS
	2 K	2560 × 1440	60/30 FPS
	720 P	1280 × 7201	20/60/30 FPS
	1080 P	1920 × 1080	120/60/30 FPS

**Table 2:** Comparison of our proposed model with mainstream models.

Dataset	Models	Precision	Recall	F1 Score	${\mathrm{mAP}}_{@0.5}$	Parameters	FLOPs	Size
*S. dumerili*	Faster RCNN	77.2%	78.4%	77.8%	82.3%	137.1 M	370.2 G	108.0 MB
	CenterNet	79.2%	79.4%	79.3%	80.1%	32.7 M	70.2 G	124.0 MB
	RetinaNet	81.0%	60.5%	69.3 %	56.2%	37.9 M	170.1 G	108.0 MB
	SSD	84.4%	89.5%	86.9%	87.0%	18.4 M	15.5 G	90.6 MB
	YOLOv4-tiny	83.7%	73.4%	80.3%	84.2%	6.1 M	**6.9 G**	22.4 MB
	YOLOv5s	90.6%	87.8%	89.2%	93.3%	**2.6 M**	7.7 G	7.7 MB
	YOLOX-s	90.2%	89.6%	89.9%	94.4%	8.9 M	26.6 G	34.3 MB
	YOLOv7	88.5%	89.0%	88.8%	92.4%	37.2 M	105.1 G	74.3 MB
	YOLOv8n	89.2%	88.4%	88.8%	92.2%	3.1 M	8.1 G	**6.0 MB**
	SD-YOLOv8	**93.3%**	88.9%	**91.0%**	**95.7%**	3.5 M	12.7 G	7.6 MB
Real-world	Faster RCNN	66.4%	**75.4%**	70.6%	71.5%	137.1 M	370.2 G	108.0 MB
	CenterNet	67.8%	66.7%	67.2%	**76.2%**	32.7 M	70.2 G	124.0 MB
	RetinaNet	62.4%	65.0%	63.7%	56.7%	37.9 M	170.1 G	108.0 MB
	SSD	70.6%	64.9%	67.6%	71.8%	26.3 M	62.7 G	94.1 MB
	YOLOv4-tiny	75.3%	61.5%	67.7%	66.4%	6.4 M	**6.5 G**	22.4 MB
	YOLOv5s	70.4%	66.2%	68.2%	75.0%	6.1 M	6.9 G	27.2 MB
	YOLOX-s	81.5%	54.7%	65.5%	69.8%	8.9 M	26.8 G	34.3 MB
	YOLOv7	70.0%	64.8%	67.3%	64.1%	37.6 M	106.5 G	142.0 MB
	YOLOv8n	78.5%	65.7%	71.5%	69.5%	**3.1 M**	8.1 G	7.6 MB
	SD-YOLOv8	**81.6%**	66.5%	**73.1%**	73.9%	3.7 M	12.2 G	**7.6 MB**

**Table 3:** Effects of module ablations on the experimental results.

Models	Precision	Recall	F1 Score	${\mathrm{mAP}}_{@0.5}$	${\mathrm{mAP}}_{@0.5:0.95}$
YOLOv8n	89.2%	88.4%	88.8%	92.5%	66.5%
YOLOv8n + Structure	90.2%	88.5%	89.4%	94.4%	67.9%
Structure + C2f_DCN	90.1%	88.8%	89.4%	94.8%	68.9%
Structure + BAM	89.0%	88.7%	88.8%	94.6%	67.6%
Structure + SPPF_LSKA	91.1%	88.0%	89.5%	94.6%	67.2%
Structure + C2f_DCN + BAM	89.6%	87.6%	88.6%	94.6%	68.2%
Structure + C2f_DCN + SPPF_LSKA	90.6%	87.2%	88.9%	94.4%	68.1%
Structure + BAM + SPPF_LSKA	90.8%	87.0%	88.9%	94.4%	67.7%
Structure + C2f_DCN + BAM	**93.3%**	**88.9%**	**91.0%**	**95.7%**	**69.7%**
+ SPPF_LSKA (Ours)					

**Table 4:** Effects of loss function ablations on the experimental results.

Method	Precision	Recall	F1 Score	${\mathrm{mAP}}_{@0.5}$	${\mathrm{mAP}}_{@0.5:0.95}$
GIoU	91.7%	89.5%	90.4%	94.7%	69.6%
CIoU	90.5%	87.6%	89.0%	94.3%	68.5%
EIoU	91.0%	89.5%	90.2%	94.8%	68.7%
SIoU	90.7%	88.5%	89.4%	94.8%	68.7%
MPDIoU	91.4%	87.1%	89.2%	94.6%	68.6%
InnerIoU	91.8%	**89.8%**	90.8%	95.3%	69.1%
Inner-MPDIoU (Ours)	**93.3%**	88.9%	**91.0%**	**95.7%**	**69.7%**

## Data Availability

The data in this study are available upon request from the corresponding author.
